# A mixed-methods approach for understanding cancer center catchment area needs: An example of rural cancer disparities, needs, and resiliencies

**DOI:** 10.1007/s10552-026-02166-9

**Published:** 2026-04-15

**Authors:** Stephanie Evett, Amanda Kahl, Megan E. Schmidt, Kelly Wells Sittig, Meredith Meyer, Mary Charlton, Natoshia M. Askelson, Sarah H. Nash

**Affiliations:** 1 University of Iowa College of Public Health, 145 N Riverside Dr, Iowa City, IA 52242 USA; 2 State Health Registry of Iowa, College of Public Health, University of Iowa, Iowa City, IA USA; 3 University of Iowa Health Care Holden Comprehensive Cancer Center, University of Iowa, Iowa City, IA USA; 4 Iowa Cancer Consortium, Iowa City, IA USA

**Keywords:** Comprehensive cancer center, Community outreach and engagement, Rural–urban differences, Cancer surveillance, Qualitative research

## Abstract

**Purpose:**

National Cancer Institute-designated Cancer Center Community Outreach and Engagement (COE) teams are expected to collect data to describe their catchment area’s cancer burden. University of Iowa Health Care Holden Comprehensive Cancer Center employed a mixed-methods approach to describe rural–urban differences.

**Methods:**

We conducted descriptive analyses of cancer registry data to characterize cancer incidence, mortality and five-year survival in our cancer center’s catchment area. We used secondary data sources to identify cancer risk/protective factors. All analyses were stratified by rurality using Rural-Urban Continuum Codes. Rural cancer leaders were interviewed to gain local perspectives on rural cancer burden and resiliencies.

**Results:**

Rural counties had higher incidence compared to urban counties for colorectal cancer, bladder cancer, and leukemia, and lower incidence for liver and breast cancers. Rural residents had higher colorectal cancer mortality and lower liver cancer mortality. Rural residents experienced higher survival from breast and bladder cancers and lower survival from lung, pancreatic, and ovarian cancers, and myeloma. Compared to urban residents, rural residents were less likely to engage in cancer screening and human papillomavirus vaccination. They were more likely to smoke cigarettes and report no leisure time physical activity. Interviews revealed rural cancer needs related to transportation, accessible care, and education.

**Conclusion:**

The approach that this COE team used to assess its catchment area’s cancer burden may be an informative approach for other cancer centers to understand their own catchment areas. The data collected will inform policies and direct cancer center research and clinical practices to reduce Iowa’s cancer burden.

**Supplementary Information:**

The online version contains supplementary material available at 10.1007/s10552-026-02166-9.

## Introduction

National Cancer Institute (NCI)-designated Cancer Centers are expected to serve their self-defined catchment areas [1, 2]; this includes the regular collection and collation of data to describe the cancer burden and cancer risk and protective factors throughout the catchment area. This responsibility primarily belongs to the center’s Community Outreach and Engagement (COE) team [1, 2]. In collaboration with cancer center community advisory boards (CABs), COE teams and cancer center leadership can use catchment area research to inform the development of strategic plans and drive cancer center research priorities to reduce the burden of cancer. Recently, there has been some interest by cancer centers in formalizing the process to engage with and understand community needs. For example, some cancer centers have published mixed-methods approaches for undertaking comprehensive catchment area needs assessments to characterize the burden of cancer in their catchment areas [3, 4]. Here, we apply similar methods to understand the cancer burden in our catchment area.

To add to the growing body of literature around catchment area data science, we explain our methods for conducting a mixed-methods sub-population catchment area data analysis focused on rural cancer disparities in Iowa. We chose this initial focus due to Iowa’s large rural population and our interest in investigating the local impact of rural–urban cancer differences that have been observed in national studies [5]. We describe the cancer burden in rural Iowa, including secondary analysis of cancer registry and publicly available risk/protective factor data. This is supplemented with primary qualitative data collection, consisting of semi-structured interviews with key rural-serving partners. The goal of this study was both to discuss the cancer burden among rural Iowans relative to urban Iowans and to provide a roadmap of methods that other cancer center COE teams can apply to the study of their own catchment areas.

## Methods

### Catchment area

The catchment area for University of Iowa Health Care Holden Comprehensive Cancer Center is the entire state of Iowa. Iowa is a relatively rural state, with 38% of its 3.2 million residents living in a rural county [6]. Most of the population in Iowa identifies as White, at 89.8% [7]. Black or African American residents represent 4.4% of Iowa’s population, 2.8% identify as Asian, 0.6% identify as American Indian or Alaska Native, 2.2% identify as two or more races, and 6.9% identify as being of Hispanic or Latino ethnicity [7]. Nearly one-fifth of the state’s population is over the age of 65 years (18.3%) [7]. Iowa has the second highest incidence rate for all cancers combined in the US [8, 9]. It is one of only two states in the US that have a rising cancer incidence rate and is the only state that has had a rising incidence rate for the past three 5-year reporting periods [10]. It is estimated that 40% of Iowans will be diagnosed with cancer in their lifetime [11].

### Defining rurality

Rurality was defined using 2013 Rural–Urban Continuum Codes (RUCC) from the US Department of Agriculture [12]. RUCC uses county population size and proximity to metropolitan areas to categorize counties. RUCC 1–3 counties were categorized as urban, while RUCC 4–9 counties were categorized as rural.

### Secondary data sources

Cancer surveillance data were obtained from the Iowa Cancer Registry within the National Cancer Institute’s Surveillance, Epidemiology, and End Results (SEER) database to assess the burden of cancer in rural Iowa [13, 14]. Additional incidence data were obtained from the Centers for Disease Control and Prevention’s US Cancer Statistics Public Use Database, which includes data on cancer incidence and population for all 50 states, the District of Columbia, and Puerto Rico [15, 16].

Additional secondary data were obtained from the Behavioral Risk Factor Surveillance System (BRFSS), and Iowa’s Immunization Registry Information System (IRIS) [17]. The BRFSS collects state-level data about health-related risk behaviors, chronic health conditions, and the use of preventive services [18]. County-level age-adjusted BRFSS data were obtained from CDC PLACES, which provides small-area estimation for counties [19]. We used 2020 and 2021 age-adjusted BRFSS data stratified by urban and rural counties based on 2013 RUCC to examine the following risk/protective factors: reporting no leisure time physical activity (among adults aged 18 years and older), fruit/vegetable consumption less than once per day, cervical cancer screening (among adult women aged 21–65 years), mammography use (among women aged 50–74 years), colorectal cancer screening (among adults aged 50–75 years), binge drinking (among adults aged 18 years and older), current smoking (among adults aged 18 years and older), and obesity (among adults aged 18 years or older). IRIS data were used to assess HPV vaccination use. The prevalence of 2023 HPV series initiation and HPV series completion for 13–15-year-olds was calculated using 2023 Census populations.

### Statistical analysis of quantitative data

Differences in the BRFSS data risk and protective factors between the Rural–Urban Continuum Code 2013 group means were tested using ANOVA for continuous variables, and Chi-squared tests for categorical variables. Age-adjusted incidence data from 2015 to 2019 was extracted for Iowa for each cancer site using the International Classification of Diseases for Oncology, Third Edition (ICD-O-3)/World Health Organization 2008 SEER Site Recode variable. Cancer sites were ranked by highest to lowest age-adjusted incidence rate in Iowa. Age-adjusted incidence rates for the top 20 sites in Iowa were stratified by rural–urban residence status, using the dichotomized RUCC 2013 variable as described above. States’ age-adjusted incidence rates (2015–2019) were ranked from highest to lowest for the top 20 sites, identifying where Iowa ranked in comparison with other states nationally using the US Cancer Statistics. The top 20 incident cancer sites were then analyzed for rural–urban differences in age-adjusted mortality; Iowa mortality data from 2015–2019 were extracted using the Cause of Death Recode variable and analyzed by rural–urban status using the dichotomized RUCC 2013 variable. Incidence Rate Ratios and Mortality Rate Ratios were calculated.

Finally, the top 20 incident cancer sites were examined for rural–urban differences in 5-year age-standardized relative survival ratios using data from 2014 to 2019. The actuarial method was used to calculate observed survival, and the Ederer II method to calculate cumulative expected survival. Age standardization using the international cancer survival standard population 1 was used for all sites except for thyroid, cervix uteri, brain and other nervous system for which the international cancer survival standard population 2 was used, and testes, for which the international cancer survival standard population 3 was used. SEER provides different age-standardized populations to allow for the comparison of survival rates across different age distributions and time periods for more accurate comparisons [20]. Significant differences in survival were indicated using z-scores. Life tables were matched on age, sex, socioeconomic status, and race.

All analyses of cancer registry data were conducted using SEER*Stat software (version 8.4.1) [21]. Age-adjusted BRFSS data and HPV vaccination descriptive statistics were calculated using SAS 9.4 software (SAS Institute, Cary, NC).

### Qualitative interviews

Using the cancer center COE team’s diverse network of community partners and CAB members, potential interview participants from across the state were identified and emailed about the purpose of the interview to gauge interest in participating. We identified 16 leaders in rural cancer prevention and control in the state of Iowa, and 14 agreed to be interviewed (response rate = 87.5%). Interviewees consisted of representatives living and/or working in rural communities in Iowa and included individuals working in rural health clinics, non-profits, public health departments, rural health associations, and research labs. Between March and May 2023, we conducted semi-structured interviews with these individuals to identify rural cancer needs and priorities. We developed the interview guide by adapting questions originally developed for community health needs assessments [22–24]. Interview questions included those on needs or issues in rural communities regarding cancer care, resources rural communities have relating to cancer care, and any potential solutions that may improve the health or quality of life for rural residents with cancer (see Appendix). Interviews were conducted via Zoom and lasted approximately 30 minutes. Interviews were recorded and transcribed verbatim by Rev, a third-party transcription service. The University of Iowa Institutional Review Board deemed this study not human subjects research.

### Qualitative data analysis

A rapid qualitative analysis was performed on transcripts by one member of the COE team (SE) to extract themes from interviews [25]. A simple codebook was created from the interview guide and transcripts were coded to identify main themes relating to the questions asked in the interviews.

## Results

### Cancer burden in rural Iowa

Table 1 presents the top 20 leading incident cancers among Iowans for 2015–2019, stratified by rurality. Breast, prostate, colorectal, lung and uterine cancers were the top 5 incident cancer sites in the state. Iowa ranked in the top 10 states nationally for 8 of the top 20 cancer sites: oral cavity and pharynx (ranked #1), leukemia (ranked #2), esophagus (ranked #4), melanoma (ranked #5), colorectal (ranked #7), lymphoma (ranked #8), kidney and renal pelvis (ranked #8), and male testis (ranked #8). Table 1 also gives incidence and mortality rate ratios comparing rural and urban counties. Rural counties had higher incidence of colorectal and urinary bladder cancers and leukemia, and lower incidence of breast and liver cancers. Rural counties had higher mortality from colorectal cancer, and lower mortality from liver cancer than urban counties.

**Table 1:** Incidence and mortality rates for Iowa’s top 20 incident cancers, 2015–2019, Iowa Cancer Registry

Cancer site	Incidence ranking within Iowa	Iowa’s national ranking for incidence	Incidence rate per 100,000		Incidence rate ratio	Mortality rate per 100,000		Mortality rate ratio
			Rural counties	Urban counties		Rural counties	Urban counties	
Breast^a^	1	15	128.32	139.95	**0.92**	17.69	18.89	0.93
Prostate^b^	2	16	121.06	118.82	1.04	20.06	20.28	0.99
Lung and bronchus	3	16	62.57	62.38	1.00	40.44	39.65	1.02
Colorectal	4	7	44.52	40.42	**1.10**	14.70	13.26	**1.11**
Uterine and endometrial^a^	5	11	30.97	30.82	1.00	4.52	5.41	0.84
Melanoma of the skin	6	5	30.27	29.48	1.03	2.53	2.59	0.98
Lymphoma	7	8	24.6	23.29	1.06	6.43	5.86	1.10
Urinary bladder	8	12	22.68	21.21	**1.07**	4.05	4.21	0.96
Kidney and renal pelvis	9	8	20.7	19.49	1.06	4.3	4.01	1.07
Leukemia	10	2	17.93	16.24	**1.10**	6.76	6.21	1.09
Thyroid	11	20	14.71	14.56	1.01	0.58	0.52	1.11
Oral cavity and pharynx	12	1	14.79	13.87	1.07	2.65	2.53	1.05
Pancreas	13	17	14.18	13.21	1.07	11.49	11.12	1.03
Ovary^a^	14	36	9.27	10.13	0.92	6.8	7.07	0.96
Cervix Uteri^a^	15	25	7.7	7.69	1.00	1.81	1.71	1.06
Myeloma	16	14	7.06	7.5	0.94	3.39	3.33	1.02
Testis^b^	17	8	7.09	7.13	0.99	0.34	0.28	1.25
Liver & intrahepatic bile duct	18	42	6.62	7.47	**0.89**	5.17	5.92	**0.87**
Brain and other nervous system	19	16	6.76	7.03	0.96	5.24	4.9	1.07
Esophagus	20	4	5.72	5.75	0.99	4.69	4.39	1.07

^a^female only

^b^male only

^c^bolding indicates ratios that were considered significantly different

Table 2 provides 5-year age-standardized relative survival ratios for the 20 leading incident cancer sites. Compared to urban counties, rural counties had lower survival from lung, pancreatic, and ovarian cancers and myeloma, and higher survival from breast and urinary bladder cancers.

**Table 2:** Five-year age-standardized relative survival ratios for Iowa’s leading incident cancers, 2014–2019, Iowa Cancer Registry

Cancer site	Incidence ranking within Iowa	5-year relative survival (%)	
		Rural counties	Urban counties
Breast^a^	1	**93.8**	**92.0**
Prostate^b^	2	95.7	96.0
Lung and bronchus	3	**21.8**	**24.8**
Colorectal	4	67.0	65.1
Uterine and endometrial^a^	5	85.3	84.9
Melanoma of the skin	6	92.0	90.3
Lymphoma	7	73.5	77.4
Urinary bladder	8	**82.2**	**75.7**
Kidney and renal pelvis	9	74.4	78.2
Leukemia	10	65.8	65.5
Thyroid	11	96.8	97.7
Oral cavity and pharynx	12	73.4	71.2
Pancreas	13	**10.3**	**11.5**
Ovary^a^	14	**39.7**	**45.3**
Cervix Uteri^a^	15	+	74.0
Myeloma	16	**55.8**	**57.6**
Testis^b^	17	+	+
Liver & intrahepatic bile duct	18	+	15.1
Brain and other nervous system	19	+	37.7
Esophagus	20	21.7	25.9

^a^female only

^b^male only

^c^bolding indicates relative survival ratios that were considered significantly different

+ this statistic could not be calculated; z-scores were considered not valid where one or more ratio could not be calculated

### Cancer risk and protective factors in rural Iowa

Table 3 provides information on risk and protective factor prevalence in rural and urban counties. Residents in rural counties were significantly less likely than residents in urban counties to be screened for cervical cancer (82.7% and 84.0% respectively, *p* < 0.0001), colorectal cancer (70.1%, and 72.5%, *p* < 0.0001), or breast cancer (73.5% and 75.3%, *p* < 0.0001). HPV vaccination prevalence was significantly lower for series completion in rural counties compared to urban counties (47.3% and 57.7%, *p* < 0.0001).

**Table 3:** Age-adjusted prevalence of cancer risk and protective factors, stratified by rurality, 2020–2021, Iowa Behavioral Risk Factor Surveillance System

Risk/protective factor	Rural counties	Urban counties	*p*-value
No leisure time physical activity among adults ≥ 18 years	**24.3%**	**22.0%**	** < 0.0001**
Fruit consumption less than once per day	39.7%	39.5%	0.9177
Vegetable consumption less than once per day	**23.6%**	**20.5%**	**0.005**
Cervical cancer screening among women 21–65 years	**82.7%**	**84.0%**	** < 0.0001**
Mammography among women aged 50–74 years	**73.5%**	**75.3%**	** < 0.0001**
Colorectal cancer screening among adults aged 50–75 years	**70.1%**	**72.5%**	** < 0.0001**
HPV initiation rates	18.7%	17.7%	0.3271
HPV series completion rates	**57.7%**	**47.3%**	** < 0.0001**
Binge drinking among adults ≥ 18 years	23.1%	23.2%	0.8535
Current smoking among adults ≥ 18 years	**18.5%**	**16.4%**	** < 0.0001**
Obesity among adults ≥ 18 years	**37.7%**	**36.5%**	**0.045**

^a^bolding indicates differences were considered statistically significant

Residents of rural counties were significantly more likely than those in urban counties to report consuming vegetables less than once per day (23.6% and 20.5%, *p* = 0.005). Smoking cigarettes was significantly more often reported among residents of rural counties compared to urban counties (18.5% and 16.4%, *p* < 0.0001). Rural counties had a significantly higher proportion of people with obesity compared to urban counties (37.7% and 36.5%, *p* = 0.045). There were no significant differences in the prevalence of binge drinking (23.1% and 23.2%, *p* = 0.8535), consumption of fruit less than once per day (39.6% and 39.5%, *p* = 0.9177), and HPV series initiation (18.7% and 17.7%, *p* = 0.3271) between rural and urban counties.

### Cancer needs and barriers to care among rural Iowans

The top needs for Iowa’s rural cancer patients, as identified by interview participants, were transportation, accessible care, and education (Table 4).

**Table 4:** Top barriers to cancer care in rural Iowa

Need	
Transportation	• Long travel times • Difficult to utilize and navigate public transit to travel across counties for care
Accessible care	• Lack of cancer care services in rural communities (e.g., clinical trials, radiation) • Long wait times for local care • Healthcare workforce shortages
Education	• Lack of awareness about cancer risk factors (e.g., smoking) • Lack of awareness about protective factors (e.g., cancer screenings, regularly seeing a primary care provider) • Greater belief in misinformation/disinformation

Specific issues relating to transportation included long travel times and difficulties traveling across counties for care. Interviewees shared that for rural cancer patients, one single appointment would “take them out for a whole day” as the travel times to get to and from appointments were lengthy, highlighting a need for care closer to rural communities. Other participants mentioned the challenge of using public transit to travel across counties to receive care. Although most counties have public transit services available, navigating these services to travel across counties, or even between larger communities within counties, can be difficult.

Interviewees also mentioned a lack of cancer care services in rural communities, which requires rural patients to travel to receive these services. One participant noted, “*Survivorship and end-of-life care is almost non-existent in rural areas”* (Participant 2). Other participants shared the lack of clinical trials and radiation services available in rural communities. If care was available in rural communities, long wait times were mentioned as a barrier to care. More specifically, one participant shared, “*It seems like every other month, another month gets added onto the GI wait list, especially in those rural communities to be screened or have a diagnostic or screening colonoscopy. So that access to a provider and the wait time is increasingly becoming larger”* (Participant 10). Further, healthcare workforce shortages exacerbate the lack of accessible care for rural patients, as these rural communities are seeing shortages of primary care providers, medical oncologists, social workers, nurse navigators, financial navigators, dieticians, etc.

Finally, educational needs in rural communities mentioned by participants related to a lack of awareness about cancer risk and protective factors. Participants mentioned that rural community members are often unaware of major risk factors for cancer, and have higher rates of risky health behaviors, such as smoking. A lack of awareness of the importance of cancer screenings and seeing a primary care physician regularly was noted by participants as an educational deficit. Additionally, a greater belief in misinformation/disinformation in rural communities was shared by a few participants, with one participant sharing, “*I think even trying to get people to wear sunscreen, it’s like this conspiracy theory now”* (Participant 6).

### Assets and resources to address barriers in rural communities

Top resources and assets to address cancer care barriers in rural communities included transportation and lodging resources, educational resources, and healthcare resources (Table 5). Resources relating to transportation and lodging included programs through the American Cancer Society, such as Road to Recovery and the Hope Lodge, as well as the organization Above + Beyond Cancer, a non-profit based in Des Moines that provides a number of services including overnight accommodations in hotels to cancer patients traveling to receive treatment. Other resources included gas cards, local community health workers driving patients to and from appointments, and partnerships between local cancer centers and hotels to offer free or reduced rates for cancer patients traveling to receive care.

**Table 5:** Top assets to address cancer care barriers in rural Iowa

Asset	
Transportation & lodging resources	• American Cancer Society (Road to Recovery, Hope Lodge) • Above + Beyond Cancer • Gas cards • Local community health workers driving patients • Partnerships between local cancer centers and hotels
Educational resources	• Education from healthcare providers on importance of cancer screening • Educational sessions at local libraries • North Iowa Addiction Prevention Alliance • American Cancer Society National Roundtable discussions
Healthcare resources	• Local cancer screening services • Local hospitals, community health centers, and local public health departments • Iowa Oncology Navigation Network • Collaborative Care Consults

In terms of educational resources, participants mentioned that healthcare providers educate patients about the importance of cancer screening and hold community-wide educational sessions at libraries. The North Iowa Addiction Prevention Alliance and the American Cancer Society National Roundtable discussions were both highlighted as educational resources for rural communities.

Finally, when talking about healthcare resources, several participants mentioned that some screening services were available in rural communities. Local hospitals, community health centers, and local public health departments were also mentioned as key healthcare resources as they can provide care and services close to home for rural residents. The Iowa Oncology Navigation Network, housed within the Iowa Cancer Consortium to connect patient navigators in the state, and Collaborative Care Consults, which allow rural oncologists to collaborate with oncologists at University of Iowa Health Care via telehealth, were other resources mentioned in this domain.

### Possible solutions

Participants proposed several possible solutions to identified barriers (Table 6). Those relating to the top needs include increased funding and volunteers for ride-share programs, such as Road to Recovery, and partnerships with public transit to help get patients to and from appointments. To improve access to care, some participants mentioned using community health workers to fill gaps in services, particularly around case management, and incentivizing healthcare providers to practice in rural communities. Educational programs focusing on the importance of cancer screening and regular primary care were proposed to address the educational deficits in rural communities.

**Table 6:** Potential solutions to address cancer care barriers in rural Iowa

Solution	
Transportation	• Increase funding and volunteers for ride-share programs • Partner with public transit to transport patients to and from appointments
Accessible care	• Utilize community health workers • Incentivize healthcare providers to practice in rural communities
Education	• Develop programs focused on the importance of protective factors (e.g., cancer screenings, regular primary care)

## Discussion

In this study, we describe a mixed-methods process for understanding cancer center catchment area needs and disparities, using an example of cancer among rural residents in Iowa. We used a combination of publicly available data on cancer incidence, mortality, survival and cancer risk and protective factors, as well as primary qualitative data collection to understand barriers, resources, and solutions in more detail. Together, these data indicate potential research and clinical priorities for the cancer center, which may include cancers for which we observed disparities affecting rural populations across incidence, mortality and survival (e.g., colorectal cancer, lung cancer, ovarian cancer); risk factors that were more common (or protective factors that were less common) among rural residents (e.g., smoking, physical inactivity, vegetable intake, lower cancer screening); and barriers experienced by rural residents to cancer screening and care (e.g., transportation, lack of accessible cancer care, and lack of education around cancer). Further, these data indicate resources that exist in rural communities to address needs, that could serve as a foundation for cancer center activities. These data and processes have been used by Holden Comprehensive Cancer Center leadership to develop a catchment area assessment plan, as well as inform the cancer center’s strategic plan.

Several NCI designated comprehensive cancer centers have published mixed-methods approaches to characterize their catchment areas. These various approaches have utilized publicly available data [3, 4, 26, 27] in combination with interviews [3, 28], surveys [3, 26, 28], and focus groups/listening sessions [4, 27]. Using a mixed-methods approach provides a more comprehensive understanding of the issues than is possible with quantitative data alone and allows COE teams to incorporate the voices of those we serve into our catchment area assessments. Together, this growing body of literature advances the field of catchment area data science by providing examples of different methods that can be used to understand the catchment area burden. Future opportunities for the field include incorporating consideration of systems and structures in place that hinder healthcare access and challenge healthy lifestyles; and research providing examples of how COE programs can bridge the gap between data and impact.

In addition to providing an example of how cancer centers can advance their catchment area data science, our results add to a body of literature that describes differences in cancer burden and risk among rural and urban populations. Consistent with our findings, higher incidence rates in rural areas have been shown for colorectal cancer [5], urinary bladder cancer [5], and leukemia [5], while higher mortality rates in rural areas compared to urban areas have been documented for colorectal cancer [29, 30]. Lower incidence of breast and liver cancers in rural areas has also been established [5, 30]. Further, the cancer sites we identified as having lower survival in rural areas compared to urban areas (i.e., lung, pancreatic, ovarian cancers, and myeloma) are also in line with previous research [31–33], with the exception of myeloma, where findings have been mixed [34, 35]. Exact reasons for our finding of lower myeloma survival in rural areas compared to urban areas are unknown, though may be attributable to differences in myeloma risk factors, such as age, race, sex or family history [36]. Differences in myeloma survival may also relate to rural–urban differences in access to care, diagnostic services, and cancer treatment [37]. In contrast, our finding that survival from breast and urinary bladder cancer was higher in Iowa’s rural counties compared to urban counties has not been documented in other studies [38, 39]. This could be driven by differences in willingness to travel for certain treatments among Iowa’s rural residents [40]; however, further investigation into the drivers of this finding is warranted.

Differences in cancer risk between urban and rural populations are likely, at least in part, driven by differences in the prevalence of modifiable risk and protective factors that could be targets for intervention. For example, the prevalence of cigarette smoking, physical inactivity, and obesity [41] is higher in rural versus urban areas. We observed similar patterns in our data. Additionally, fruit and vegetable consumption is lower in rural areas compared to urban areas [42]. Lower vegetable consumption in rural areas is in alignment with our findings; however, we did not find a significant difference between rural and urban areas regarding fruit consumption. As shown in the present study, cancer screening prevalence is consistently lower in rural communities compared to urban communities [41], which may account for differences in stage at diagnosis for some of these cancers [43, 44]. Our finding of lower HPV vaccination rates in rural counties compared to urban counties has also been documented on a national scale [45]. Social and structural determinants of health often drive access to and engagement in these behaviors at the individual level; therefore, policies and programs to increase access to healthy foods, increase access to physical activity, and improve access to healthcare among rural residents are likely to be important to addressing these known disparities.

To address rural cancer disparities, cancer centers serving rural populations will need to build on existing resources and resiliencies to remove individual, community, and structural level barriers to healthcare access and healthy behaviors. The lack of accessible cancer care services for rural communities is established in the existing literature, including the lack of lung cancer screening centers [46], colorectal cancer screening services [47], general surgeons and radiation oncologists [48], and survivorship resources [49]. Our interviewees suggested use of community health workers and incentivizing healthcare providers to practice in rural communities as potential solutions to these issues; other solutions from the literature include mobile screening units [46], training primary care physicians to perform cancer screenings, such as colonoscopies [48], and relieving student loan debt for healthcare providers who practice in rural areas [48, 50]. Further, lack of access may drive rural residents to seek healthcare farther afield, yet transportation barriers for rural cancer patients are also well documented [46–48, 51] and can make it difficult for rural patients to access cancer screenings and treatment. Existing resources such as the American Cancer Society’s Hope Lodge program and others that provide overnight accommodations to those traveling for treatment, as well as existing ride-share and transportation programs may partially alleviate these barriers. A resource list of local programs providing transportation services may be one strategy to promote awareness and use of these programs [50]. Finally, many rural residents may not be aware of the importance of cancer screening and other healthy behaviors [46, 47, 51]; educational initiatives aimed at rural communities may improve knowledge and awareness. Putting our findings in the context of broader literature indicates that addressing barriers to healthcare and healthy behaviors in rural communities will require comprehensive, multi-sector efforts. Cancer centers may be in a position to collaborate with external partners across sectors to address these barriers.

The primary strength of our study was the use of the mixed-methods approach, which allowed us to benefit from the strengths of both qualitative and quantitative methods to gain a more comprehensive understanding of cancer burden, with a particular emphasis on rural Iowa. One limitation of our study was that we dichotomized our rural–urban analysis, rather than examining the cancer burden on a gradient. Another limitation of our work was relying on a single team member to perform the qualitative analysis, which has the potential to introduce bias to the analysis. Further, our approach was limited to cancer disparities in the context of rural–urban counties and did not take into account other potential sources of cancer disparities, such as area-level poverty. Finally, our findings are based on data from a single state, which may not be representative of broader regional or national trends.

## Conclusions

Our mixed-methods approach used by the cancer center’s COE team to understand the cancer burden, particularly in rural Iowa, may be an informative approach for other cancer centers aiming to more fully understand their catchment area and sub-population needs. Data generated from this approach can be used to inform policies, programs, and clinical practices to improve patient care, and reduce the cancer burden in catchment areas.

## Supplementary Information

Below is the link to the electronic supplementary material.

Supplementary file1 (DOCX 23 KB)

## Data Availability

The SEER data analyzed during the current study are available directly through the SEER program from the National Cancer Institute, (https://seer.cancer.gov/data-software/). The US Cancer Statistics Public Use Database analyzed during the study is available through the Centers for Disease Control and Prevention, (https://www.cdc.gov/united-states-cancer-statistics/public-use/index.html). The BRFSS data analyzed during the current study are available from the Centers for Disease Control and Prevention, (https://www.cdc.gov/brfss/). The HPV vaccine series initiation and completion data analyzed during our study are available through the Iowa Department of Health and Human Services (https://hhs.iowa.gov/data/immunization/human-papillomavirus-hpv-vaccine-data).

## Abbreviations

COE: Community Outreach and Engagement
NCI: National Cancer Institute
CAB: Community advisory board
RUCC: Rural Urban Continuum Codes
SEER: Surveillance, Epidemiology, and End Results
BRFSS: Behavioral Risk Factor Surveillance System
IRIS: Immunization Registry Information System
