# Supplementary material for: A mixed-methods approach for understanding cancer center catchment area needs: An example of rural cancer disparities, needs, and resiliencies
Source: Cancer Causes Control. 2026 Apr 15;37(5):82. doi: 10.1007/s10552-026-02166-9 (PMC13079507; doi:10.1007/s10552-026-02166-9)
Supplement: Supplementary file 1 — Supplementary file1 (DOCX 23 KB) [file 10552_2026_2166_MOESM1_ESM.docx]

**A mixed-methods approach for understanding cancer center catchment area needs: An example of rural cancer disparities, needs, and resiliencies**

Stephanie Evett, MPH^1^; Amanda Kahl, MPH^1,2^; Megan E. Schmidt, MEd, MPH^1,3^; Kelly Wells Sittig, MS^3,4^; Meredith Meyer, MPH^1^; Mary Charlton, PhD^1,2,3^; Natoshia M. Askelson, MPH, PhD^1,3^; Sarah H. Nash, MPH, PhD^1,2,3^

^1^University of Iowa College of Public Health, Iowa City IA

^2^State Health Registry of Iowa, College of Public Health, University of Iowa, Iowa City IA

^3^University of Iowa Health Care Holden Comprehensive Cancer Center, University of Iowa, Iowa City IA

^4^Iowa Cancer Consortium, Iowa City, IA

**Corresponding Author:** Sarah Nash, MPH, PhD Email: [sarah-nash@uiowa.edu](mailto:sarah-nash@uiowa.edu) Phone: 319-467-4282. Address: 145 N Riverside Dr, S418 CPHB, Iowa City, IA 52242. ORC-ID: 0000-0002-9038-6001

**For submission to:** Cancer Causes & Control

**Appendix - Interview Guide**

1. I’ve heard a little about your work already, but could you give me a brief overview to help frame today’s discussion?
2. In thinking about cancer care, what are the needs or issues you see in your community or the community you work in?

- Probe: What are some of the challenges, barriers, and/or needs rural people in Iowa face relating to cancer – anywhere from prevention, to screening, diagnosis, treatment or survivorship?

1. What resources does your community have that can be used to improve the health of community members with cancer?

- Probe: What are the strengths and resources in your community that address the health issues you’ve identified, such as groups, initiatives, services, or programs? Please name them.
- Probe: What else is needed in the community to maintain or improve health for community members with cancer?

1. If you could change one thing or offer one solution to improve the health and quality of life for residents in your community with cancer, what would it be?

That’s all the questions I have for you, is there anything else you’d like to add to anything we’ve discussed? Do you have any questions for me at this time?
